# Anti-platelet activity of panaxatriol saponins is mediated by suppression of intracellular calcium mobilization and ERK2/p38 activation

**DOI:** 10.1186/s12906-016-1160-7

**Published:** 2016-06-08

**Authors:** Hongyi Qi, Yongliang Huang, Yi Yang, Guojun Dou, Fang Wan, Wenwu Zhang, Huarong Yang, Li Wang, Chunjie Wu, Li Li

**Affiliations:** College of Pharmaceutical Sciences, Southwest University, 2 Tiansheng Road, Beibei District, Chongqing, 400716 China; Affiliated Hospital of Chengdu University of Traditional Chinese Medicine, Chengdu, 610075 Sichuan China; Huasun Group Co., Ltd., Chengdu, 610072 Sichuan China; College of Pharmacy, Chengdu University of Traditional Chinese Medicine, Chengdu, 611137 Sichuan China

**Keywords:** Panaxatriol saponin, Platelet aggregation, Intracellular calcium mobilization, p-ERK2, p-p38

## Abstract

**Background:**

Increased platelet aggregation is implicated in the pathogenesis of ischemic stroke and anti-platelet strategy may contribute to its therapy. Panaxatriol saponin (PTS), the main components extracted from *Panax notoginseng*, has been shown to be efficacious in the prevention and treatment of ischemic stroke in China. The aim of this study is to determine the anti-platelet activity and explore the underlying mechanisms.

**Methods:**

Inhibitory effect of PTS and its main ginsenosides on agonists-induced platelet aggregation was determined using rabbit or human platelets. Intracellular Ca^2+^ concentration ([Ca^2+^]*i*) mobilization was detected with fura-2/AM probe. MAPKs phosphorylation was determined by Western blotting.

**Results:**

Our results showed PTS inhibited the rabbit platelet aggregation induced by various agonists (collagen, thrombin and ADP). The three main ginsenosides (Rg1, Re and R1) existing in PTS also showed anti-platelet activity, while their combination exhibited no synergistic effect on rabbit platelet aggregation. Further study demonstrated that PTS and its main ginsenosides also exhibited inhibitory effect on human platelet aggregation. Mechanism study demonstrated that pre-treatment with PTS inhibited the agonists-induced intracellular calcium mobilization. Moreover, PTS significantly suppressed the activation of both ERK2 and p38 by the agonists via reducing the phosphorylation of ERK2 and p38.

**Conclusion:**

We proved that PTS is effective in anti-platelet aggregation, which may, at least in part, be related to the suppression of intracellular calcium mobilization and ERK2/p38 activation. This study may provide one reasonable explanation for the efficacy of PTS on the prevention and treatment of ischemic stroke.

## Background

Platelets which only exist in mammals mainly function as stopping bleeding at the site of interrupted endothelium. Under normal physiological condition, platelets are quiescent and don’t bind to fibrinogen. Once there is an injury in the endothelium, platelets will adhere to the interrupted endothelium and become activated via changing shape, turning on receptors and secreting chemical messengers. Then, they interconnect through receptor bridges to form aggregation and finally promote the blood coagulation [1]. However, abnormal platelet activation triggered by pathophysiological factors can lead to the development and progression of vascular diseases [2]. Increased platelet aggregation is believed to play an important role in the pathogenesis of ischemic stroke [3]. Therefore, anti-platelet therapy may represent an alternative way for the prevention and treatment of ischemic stroke.

Panaxatriol saponin (PTS) is one of the major components of Panax notoginseng and composed of ginsenoside Rg1 (Rg1), notoginsenoside R1 (R1) and ginsenoside Re (Re). It has been clinically used in China for the treatment of cerebral infarction. Previous pharmacological study demonstrated that PTS alleviated the focal cerebral ischemia-induced injury via reducing the cerebral edema, up-regulating HSP70 expression and down-regulating transferring [4]. Our recent investigation indicated that PTS exerted neuroprotection through modulating the cyto-protective Nrf2 signaling pathway [5]. In addition, several studies have reported that ginsenosides, for example, Rp1 [6], Rg3 [7], Rg1 [8], Rh1 and F1 [9], exhibited the anti-platelet activities. In view of the protective effect on ischemic stroke, whether PTS also has the anti-platelet activity is accordingly drawing our attention. Therefore, we designed experiments to investigate the effect of PTS on platelet aggregation and explore the underlying mechanisms in the current study.

## Methods

### Materials

PTS extracted from Panax notoginseng was obtained from Huasun Group Co., Ltd. (Sichuan, China). As determined in our previous study [5], PTS is comprised of Rg1, R1 and Re with the concentration of 46.1, 12.3 and 5.7 %, respectively. The antibodies against p-ERK, p-p38 and HRP conjugated goat anti-mouse IgG were purchased from Cell Signaling Technology (Boston, MA, USA). Thrombin and collagen were purchased from Sigma-Aldrich (St. Louis, MO, USA). ADP was obtained from Tokyo Chemical Industry (Shanghai) Co. Ltd. (Shanghai, China). Fura-2/AM was obtained from Beyotime Biotechnology Corporation (Jiangsu China). Other chemicals were obtained from Sigma-Aldrich Co. (St. Louis, MO, USA) unless indicated otherwise. Human peripheral blood was collected from healthy human donors after obtaining the informed consent under a Chengdu University of Traditional Chinese Medicine Internal Review Board–approved protocol.

### Experimental animals

New Zealand albino rabbits (2.0 ± 0.2 kg) used in this study were obtained from Experimental Animal Center of Chengdu University of Traditional Chinese Medicine. The animals were acclimated for 1 week prior to the experiments, and housed in an air-conditioned animal room with a 12/12 h light/dark cycle at a temperature of 25 ± 1 °C and humidity of 50 ± 10 %. The animals were provided with a laboratory diet and water *ad libitum*. All experimental protocols involving the use of animals were conducted in accordance with National Institutes of Health (NIH) guidelines and approved by the Committee on Animal Care at the Southwest University.

### Platelet preparation

The preparation of the platelets has been described previously with minor modifications [6]. The human blood or rabbit blood was transferred to a tube containing 1 ml of a citrate phosphate dextrose solution (90 mM Na_3_C_6_H_5_O_7_ · 2H_2_O, 16 mM C_6_H_8_O_7_ · H_2_O, 16 mM NaH_2_PO_4_ · H_2_O, 142 mM dextrose). Platelet-rich plasma (PRP) was obtained by centrifuging rabbit blood samples at 230 × g for 5 min. Platelets were precipitated by centrifugation of the PRP at 800 × g for 15 min and washed twice with the washing buffer (10 mM Na_3_C_6_H_5_O_7_ · 2H_2_O,150 mM NaCl,1 mM EDTA,1 % (w/v) dextrose and pH 7.4). The pellet was re-suspended in HEPES buffer (140 mM NaCl, 2.7 mM KCl, 3.8 mM HEPES, 5 mM EGTA and pH 7.4) and the cell dilution was adjusted to 4 × 10^8^ cells · ml^-1^ for subsequent experiment.

### Platelet aggregation assay

Platelet aggregation was performed as previously described [10]. Briefly, blank solvent and test samples at various concentrations were added to 200 μl of PRP and after 15 min aggregation was initiated by adding different inducers in each well of 96-well plates, respectively. Optical density (OD) at 630 nm was measured with Microplate Reader (BioTek ELX800) after 1, 2, 4, 10 min of the addition. All the experiments were performed in triplicate. The platelet aggregation rate is expressed as PAR (%) and calculated as follows:

$$ \mathrm{P}\mathrm{A}\mathrm{R}\kern0.5em \left(\%\right)\kern0.5em =\kern0.5em \left({1\hbox{-} \mathrm{O}\mathrm{D}}_0\right)/{\mathrm{OD}}_1\kern0.5em \times \kern0.5em 100\% $$

Whereas OD_0_ and OD_1_ indicate the optical density before and after the inducer is added, respectively.

The following equation is used to calculate the inhibition rate (IR) of platelet aggregation.

$$ \mathrm{I}\mathrm{R}\left(\%\right)\kern0.5em =\kern0.5em \left({1\hbox{-} \mathrm{P}\mathrm{A}\mathrm{R}}_{\mathrm{test}}\right)/{\mathrm{PAR}}_{\mathrm{blank}}\kern0.5em \times \kern0.5em 100\% $$

Whereas PAR_test_ and PAR_blank_ represent the PAR of the tested samples and the blank solvent, respectively.

### Determination of the intracellular calcium concentration ([Ca^2+^]_i_)

[Ca^2+^]_*i*_ was determined with fura-2/AM as described previously [6]. Briefly, the PRP with concentration of 4 × 10^8^ cells/ml was incubated with 5 *μ*M of fura-2/AM for 60 min at 37 °C. The fura-2-loaded washed platelets were pre-incubated with the tested sample or the blank solvent (saline) for 3 min at 37 °C in the presence of 1 mM CaCl_2_. Next, the platelets were stimulated with thrombin (0.1 U · ml^-1^), collagen (2.5 *μ*g · ml^-1^) or ADP (10 *μ*M) for 5 min, respectively. Fura-2 fluorescence was measured in a spectrofluorometer (F-2500, Hitachi, Japan) with an excitation wavelength of 340 nm and an emission wavelength of 510 nm. The [Ca^2+^]_*i*_ was calculated by the method of Schaeffer [11]: [Ca^2+^]_*i*_ in cytosol = 224 nM×(*F* − *F*_*min*_)/(*F*_*max*_ − *F*), where 224 nM is the dissociation constant of the fura-2-Ca^2+^ complex, and *F*_*min*_ and *F*_*max*_ represent the fluorescence intensity levels at very low and very high Ca^2+^ concentrations, respectively. In our experiment, *F*_*max*_ is the fluorescence intensity of the fura-2-Ca^2+^ complex at 510 nm after the platelet suspension containing 1 mM of CaCl_2_ had been solubilized by Triton X-100 (0.1 %). *F*_min_ is the fluorescence intensity of the fura-2-Ca^2+^ complex at 510 nm, after the platelet suspension containing 20 mM Tris/3 mM of EGTA had been solubilized by Triton X-100 (0.1 %). *F* represents the fluorescence intensity of the fura-2-complex at 510 nm after the platelet suspension was stimulated by inducer, with and without the tested samples, in the presence of 1 mM CaCl_2_.

### Western blotting analysis

The cellular proteins were extracted and analyzed for protein expression as previously described [12]. Briefly, thirty micrograms of the cellular proteins were resolved by electrophoresis in 10 % SDS-polyacrylamide gel, and subsequently transferred to polyvinylidene difluoride (PVDF) membrane. Following 1 h incubation in a fresh TBS buffer containing 0.1 % Tween-20 and 5 % BSA, the blots were probed with specific antibodies including anti-p-ERK, anti-p-p38 and anti-*β*-actin. The bound primary antibodies were detected by HRP conjugated anti-mouse IgG accordingly. The activity of peroxidase on the blot was visualized by enhanced chemiluminescence (ECL) detection reagents (GE Healthcare, Sweden).

### Statistical analysis

All data were presented as mean ± SD from three independent experiments. Statistical analysis was performed by two-tail Student’s *t*-test. A *P*-value of less than 0.05 was considered to be statistically significant.

## Results

### Inhibitory effect of PTS on agonist induced rabbit platelet aggregation

**Fig. 1:**
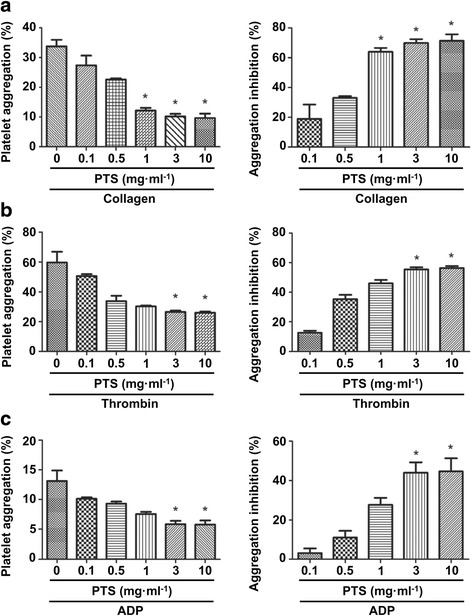
Inhibitory effect of PTS on collagen, thrombin or ADP induced platelet aggregation. Platelets (4 × 10^8^ cells · ml^-1^) were pre-incubated with or without PTS and then stimulated with **a** collagen (2.5 *μ*g · ml^-1^), **b** thrombin (0.1 U · ml^-1^) or **c** ADP (10 *μ*M). The platelet aggregation rate and the inhibition rate of platelet aggregation were determined. Bar graphs show mean ± SD of at least 3 independent experiments performed. *p < 0.05 vs. agonist activated control

To determine the effect of PTS on rabbit platelet aggregation, we examined its anti-platelet activity against stimulation of different agonists. washed platelets were pre-incubated with different concentrations of PTS and then exposed to collagen, thrombin and ADP, respectively. As shown in Fig. 1a, PTS significantly inhibited the rabbit platelet aggregation activated by collagen at the concentration of 1, 3 and 10 mg · ml^-1^, with the inhibition rate of 63.9, 69.8 and 71.3 %, respectively. Figure 1b shows that treatment of rabbit platelets with 3 and 10 mg · ml^-1^ PTS markedly inhibited the aggregation induced by thrombin, with the inhibition rate of 55.5 and 56.4 %, respectively. Similarly, 3 and 10 mg · ml^-1^ PTS also significantly inhibited ADP-induced rabbit platelet aggregation with the inhibition rate of 55.3 and 55.9 %, respectively.

### Inhibitory effect of the main ginsenosides in PTS on thrombin induced rabbit platelet aggregation

**Fig. 2:**
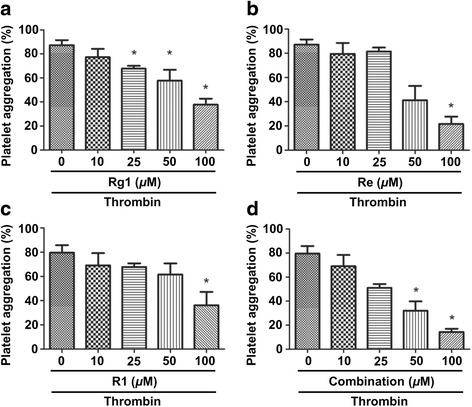
Inhibitory effect of the main ginsenosides in PTS on thrombin induced platelet aggregation. Platelets (4 × 10^8^ cells · ml^-1^) were pre-incubated with or without Rg1 (**a**), Re (**b**), R1 (**c**) or their combination of equal concentration (**d**), and then stimulated with thrombin (0.1 U · ml^-1^). The platelet aggregation rate was determined. Bar graphs show mean ± SD of at least 3 independent experiments performed. *p < 0.05 vs. agonist activated control

As PTS consists of three main ginsenosides (Rg1, Re and R1), we are then interested in the effect of these single compounds on rabbit platelet inhibition and asked whether there is a synergistic effect when they are used as a combination. As shown in Fig. 2a, Rg1 dose-dependently inhibited the aggregation activated by thrombin within the concentration of 10-100 *μ*M. However, both Re and R1 only significantly inhibited the thrombin induced rabbit platelet aggregation at 100 *μ*M within the same tested concentration range (Fig. 2b and c). When these three compounds were used together at the same concentration, only the combination with 50 and 100 *μ*M showed significantly inhibitory activity on thrombin induced rabbit platelet aggregation and there was no synergistic effect observed.

### Inhibitory effect of PTS and its main ginsenosides on agonist induced human platelet aggregation

**Fig. 3:**
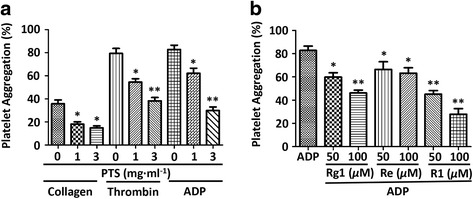
Inhibitory effect of PTS and its main ginsenosides on human platelet aggregation. **a** Human platelets (4 × 10^8^ cells · ml^-1^) were pre-incubated with or without PTS and then stimulated with collagen (2.5 *μ*g · ml^-1^), thrombin (0.1 U · ml^-1^) or ADP (10 *μ*M). The platelet aggregation rate was determined. **b** Human platelets (4 × 10^8^ cells · ml^-1^) were pre-incubated with or without Rg1, Re, R1, and then stimulated with ADP (10 *μ*M). The platelet aggregation rate was determined. Bar graphs show mean ± SD of at least 3 independent experiments performed. *p < 0.05, **p < 0.01 vs. agonist activated control

To further confirm the anti-platelet aggregation effect of PTS and its main ginsenosides, we then determined their inhibitory effect on human platelet aggregation. As shown in Fig.3a, PTS significantly inhibited the human platelet aggregation induced by collagen, thrombin and ADP, respectively. Moreover, the three main ginsenosides (Rg1, Re and R1) also exhibited significant anti-platelet aggregation effect against ADP-induced human platelet aggregation (Fig.3b).

### Effect of PTS on calcium mobilization

**Fig. 4:**
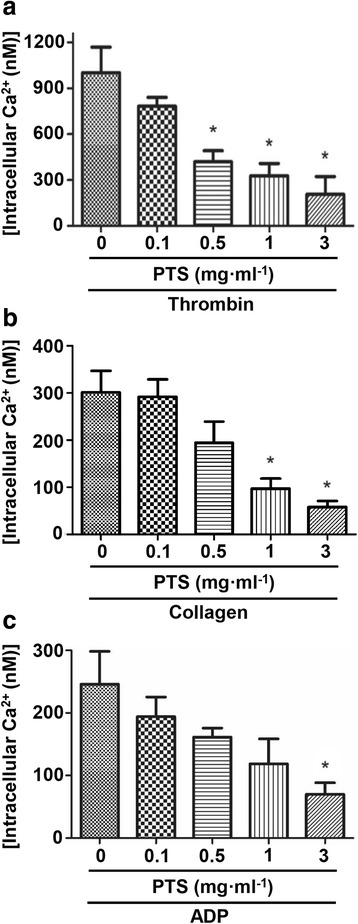
Effect of PTS on intracellular Ca^2+^ concentration ([Ca^2+^]*i*) in thrombin, collagen or ADP activated platelets. Platelets were loaded with Fura-2/AM as described in ‘Materials and Methods Section’. The platelets (4 × 10^8^ cells · ml^-1^) were pre-incubated with or without a PTS and then the platelets were stimulated with thrombin (0.1 U/ml) (**a**), collagen (2.5 *μ*g · ml^-1^) (**b**) or ADP (10 *μ*M) (**c**) for 5 min, respectively. [Ca^2+^]*i* levels were determined as described in ‘Materials and Methods Section’. Bar graphs show mean ± SD of at least 3 independent experiments performed. *p <0.05 vs. agonist activated control

It’s well established that [Ca^2+^]_*i*_ is critical for the activation and aggregation of platelet aggregation [13–15]. The decrease of [Ca^2+^]_*i*_ directly inhibits the platelet aggregation. Therefore, we determined the influence of PTS on the calcium mobilization. Figure 4a showed that thrombin alone markedly enhanced the [Ca^2+^]_*i*_. When pre-incubation of platelets with various concentration of PTS, the [Ca^2+^]_*i*_ induced by thrombin dose-dependently decreased by 58.2, 67.3 and 79.4 % at concentrations of 0.5, 1 and 3 mg · ml^-1^ (*p* < 0.05), respectively. Similarly, Fig. 4b and c show that 1 and 3 mg · ml^-1^ of PTS significantly reduced collagen-activated [Ca^2+^]_*i*_ and 3 mg · ml^-1^ of PTS significantly reduced ADP-activated [Ca^2+^]_*i*_.

### Effect of PTS on the phosphorylation of MAPKs

**Fig. 5:**
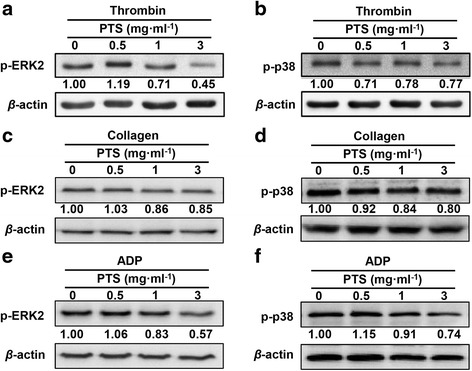
Effect of PTS on the phosphorylation of ERK2 (**a**) and p38 (**b**) in the agonists-activated platelets. Washed platelets (4 × 10^8^ cells · ml^-1^) were pre-incubatted with vehicle or PTS at the concentrations indicated prior to stimulation with thrombin (0.1 U/ml) (A and B), collagen (2.5 *μ*g · ml^-1^) (**c** and **d**) or ADP (10 *μ*M) (**e** and **f**). Proteins were extracted, separated by SDS-PAGE transferred to PVDF and immunoblotted with antibodies against p-ERK, p-p38 and *β*-actin. Blots were visualized by ECL. The blots were representative of three independent experiments. The value was expressed as each normalized data relative to agonist alone

Since MAPKs (ERK2, JNK1 and p38) are reported to be present in platelets and involved in the action of numerous anti-platelet agents [16], we determined whether thrombin induced MAPKs phosphorylation is regulated as a signaling pathway in the anti-platelet activity of PTS. As shown in Fig. 5a, PTS with 1 and 3 mg · ml^-1^ significantly reduced the phosphorylation of ERK2 induced by thrombin. Meanwhile, PTS with the concentrations of 0.5-3 mg · ml^-1^ significantly reduced the phosphorylation of p38 induced by thrombin (Fig. 5b). Then, we further determined the effect of PTS on the phosphorylation of ERK2 and p38 induced by collagen and ADP, respectively. Figure 5c and e show that the phosphorylation of ERK2 induced by collagen and ADP was significantly reduced by PTS and Fig. 5d and f show that the phosphorylation of p38 induced by collagen and ADP was also significantly reduced by PTS.

## Discussion

In this study, we first determined the inhibitory activity of PTS on platelet aggregation activated by collagen, thrombin and ADP, respectively. It’s well established that the three different agonists act on different types of receptors. Collagen is a potent ligand against integrin-type receptors, including *α*2*β*1 and glycoprotein VI, whereas thrombin and ADP are potent ligands of G-protein coupled seven transmembrane receptors (GPCR) (PAR-1 and PAR-4 thrombin receptors, P2Y_1_ and P2Y_12_ ADP receptors), respectively [17–19]. Our result demonstrated that PTS showed anti-platelet activity irrelevant to the types of agonists, suggesting that PTS may not function as an antagonist to counteract the binding to platelet receptors on the plasma membrane of these three agonists. PTS is a complex fraction containing three major ginsenosides Rg1, Re and R1 [5]. Previous study showed that Rg1 [8] exhibited anti-platelet activity. Then, we are interested to determining the effect of another two main compounds (Re and R1) in PTS on platelet aggregation and whether the combination of these three ginsenosides could function synergistically. As a result, Rg1 inhibited the platelet aggregation at relative low concentrations, whereas Re showed stronger effect at higher concentration. The inhibitory activity of R1 was much similar than that of Re. However, the potency of the combination in inhibiting platelet aggregation didn’t improve, indicating there may be no synergistic effect among the components in PTS. To make our study become more clinical relevance, we then determined the effect of PTS and its main ginsenosides on human platelet aggregation. As a result, both PTS and its main ginsenosides showed significant inhibitory effect.

[Ca^2+^]_*i*_ plays an important role in the activation and aggregation of platelets and the thrombus formation. The exogenous or endogenous activation of a membrane receptor with thrombin, collagen or ADP leads to the influx of Ca^2+^ into the platelets [20, 21]. The activation of thrombin receptors PAR1 and PAR4 or a P2Y ADP receptor, which belong to the Gq protein-coupled receptors, leads to the activation of phospholipase (PLC)-*β*. Additionally, collagen binds to glycoprotein VI on the platelet surface leading to the increase of PLC activity by phosphorylating PLCγ-2 on a tyrosine residue. Then, these receptors stimulate the release of inositol trisphosphate (IP3) from phosphatidylinositol bisphosphate (PIP2). Finally, the calcium mobilization is resulted by the binding of IP3 to type 2 IP3 receptors in the secretory granules of human platelets [14, 22, 23]. To find out the possible signaling pathway involved in the downstream of membrane receptors, we determined the effect of PTS on the intracellular calcium mobilization. In our study, all the three agonists thrombin, collagen and ADP stimulate the [Ca^2+^]_*i*_. While pre-treatment with PTS, the [Ca^2+^]_*i*_ in all these agonists-activated platelets remarkably reduced, suggesting that inhibition of calcium mobilization may be one of the common signaling pathways responsible for the inhibitory effect of PTS against platelet aggregation stimulated by these three different agonists.

MAPKs including ERK2, JNK1 and p38 are present in platelets and activated by various agonists [16, 24]. Our results demonstrated that PTS significantly suppressed the activation of both ERK2 and p38 induced by thrombin, collagen or ADP via reducing the expression level of p-ERK2 and p-p38. However, PTS showed no statistically significant influence on p-JNK1 (data not shown). It’s reported that ERK2 and p38 have complementary effects in the control of platelet adhesion to collagen. In static adhesion condition, p38 was involved in platelet adhesion and spreading. In blood flow condition, p38 activation is required for platelet adhesion at low collagen coverage densities, while ERK2 activation is necessary for platelet adhesion at higher collagen coverage densities [16]. Therefore, suppression of ERK2 and p38 activation may be partially as a common signaling pathway responsible for the inhibitory effect of PTS on platelet aggregation in our study.

## Conclusion

In summary, we have investigated the potential effect of PTS on platelet aggregation and explored the underlying mechanisms in this study. PTS exhibited anti-platelet activity against stimulation of different agonists, including collagen, thrombin and ADP. The three major ginsenosides used alone also showed anti-platelet activity, whereas their combination didn’t improve the effect synergistically. Further mechanism study revealed that PTS significantly inhibited intracellular calcium mobilization induced by the three different agonists. Moreover, PTS also inhibited the phosphorylation of ERK2 and p38 induced by all the three agonists. Collectively, our study showed PTS has anti-platelet activity and inhibition of intracellular calcium mobilization and p-ERK2/p-p38 may, in part, be as the common signaling pathways responsible for this effect. The anti-platelet activity of PTS may be implicated in its beneficial effect on the prevention and treatment of ischemic stroke.

## Abbreviations

ADP, adenosine diphosphate; BSA, bovine serum albumin; ECL, enhanced chemiluminescence; EGTA, ethylene glycol tetraacetic acid; *F*_*max*_, the fluorescence intensity levels at very high Ca^2+^ concentrations; *F*_*min*_, the fluorescence intensity levels at very low Ca^2+^ concentrations; GPCR, G-protein coupled seven transmembrane receptor; HEPES, 4-(2-hydroxyethyl)-1-piperazineethanesulfonic acid; HRP, horseradish peroxidase; HSP70, heat shock protein 70; IP3, inositol trisphosphate; Nrf2, nuclear factor (erythroid-derived 2)-like 2; OD, optical density; PAR, platelet aggregation rate; PIP2, phosphatidylinositol bisphosphate; PRP, platelet-rich plasma; PTS, panaxatriol saponin; PVDF, polyvinylidene difluoride; R1, notoginsenoside R1; Re, ginsenoside Re; Rg1, ginsenoside Rg1; SDS, sodium dodecyl sulfate
